# 人肺癌细胞中*TSLC1*基因表达缺失与DNA甲基化的相关性研

**DOI:** 10.3779/j.issn.1009-3419.2010.05.16

**Published:** 2010-05-20

**Authors:** 树红 明, 静 高, 铁英 孙

**Affiliations:** 1 100730 北京，卫生部北京医院呼吸科 Department of Respiratory Medicine, Beijing Hospital Ministry of Health, Beijing 100730, China; 2 100142 北京，北京大学临床肿瘤学院、北京肿瘤医院暨北京市肿瘤防治研究所消化肿瘤内科，恶性肿瘤发病机制及转化研究教育部重点实验室 Key Laboratory of Carcinogenesis and Translational Research (Ministry of Education), Department of GI Oncology, Peking University School of Oncology, Beijing Cancer Hospital & Institute, Beijing 100142, China

**Keywords:** TSLC1, 肺肿瘤, 基因表达缺失, DNA甲基化, TSLC1, Lung neoplasms, Gene silencing, DNA methylation

## Abstract

**背景与目的:**

TSLC1在多种肿瘤中表达下调或失活，其表达下调与DNA高甲基化有很大关系。本研究旨在探索TSLC1在肺癌细胞中的表达缺失与其启动子区DNA甲基化的关系。

**方法:**

采用RT-PCR和Real-time PCR方法检测TSLC1在正常肺组织和3种肺癌细胞系（A549、NCI-H446和Calu-3）中的表达谱；运用亚硫酸氢盐修饰后测序（bisulfte sequencing）方法检测上述正常肺组织和肺癌细胞中*TSLC1*启动子区的甲基化状态；应用甲基化转移酶抑制剂5-氮-2-脱氧胞苷（5-Aza-dC）处理上述细胞后，采用Real-time PCR方法检测处理前后TSLC1的表达变化。

**结果:**

TSLC1在正常肺组织和A549细胞中表达，其启动子区DNA未发生甲基化；而在NCI-H446和Calu-3细胞中表达缺失，其启动子区DNA发生高甲基化，并且5-Aza-dC处理NCI-H446和Calu-3细胞后可促进TSLC1的表达。

**结论:**

TSLC1在肺癌细胞中的表达缺失是由其启动子区的DNA高甲基化引起。

## Abstract (en)

**Background and objective:**

The expression of TSLC1 is downregulated or abrogated in many kinds of tumors, and its downregulation is highly associated with DNA hypermethlyation. The aim of this study is to explore the relationship between TSLC1 silencing and DNA methylation of its promoter region in lung cancer cells.

**Methods:**

We detected the expression patern of TSLC1 in human normal lung tissue and three lung cancer cell lines (A549, NCI-H446 and Calu-3) by semi-quantitative RT-PCR and Real-time PCR. Ten we detected the status of DNA methylation in *TSLC1* promoter region with bisulfte sequencing in above normal lung tissue and lung cancer cell lines. Afer treatment of above cell lines with the inhibitor of DNA methyltransferase 5-Aza-2-deoxycytidine (5-Aza-dC), we detected the expression change of TSLC1 by Real-time PCR before and afer the treatment of 5-Aza-dC.

**Results:**

There was no methylation in *TSLC1* promoter region in normal lung tissue and A549 cell line in which TSLC1 expressed; while there was DNA hypermethylation in *TSLC1* promoter region in NCI-H446 and Calu-3 cell lines in which TSLC1 was abrogated, also the expression of TSLC1 in NCI-H446 and Calu-3 cell lines could be restored afer treatment of 5-Aza-dC.

**Conclusion:**

The silencing of TSLC1 in lung cancer cells is due to the hypermethylation of its promoter region.

肺癌是当今世界上发病率与死亡率极高的恶性肿瘤之一。由于缺乏有效的早期诊断方法，临床诊断的肺癌多为进展期，而且疗效很不理想。因此肺癌的早期诊断和治疗成为临床上一大难题。随着分子生物学技术的飞速发展，癌症的基因发病机制研究、基因诊断和基因治疗受到大家的关注，特别是表观遗传（epigenetic）调控的研究成为目前癌症研究的热点。早在1989年就已报道肺癌细胞中常存在DNA甲基化状态的改变^[1]^，而DNA异常甲基化是一种非基因水平改变的表观遗传调控方式^[2]^。这种现象不仅可以激活某些原癌基因的表达，而且可抑制一些抑癌基因的转录。

TSLC1（tumor suppressor in lung cancer 1）又名NECL2（nectin-like molecule 2），是正常人体组织广泛表达的免疫球蛋白样细胞粘附分子，因最初发现其在肺癌组织中表达缺失而命名为TSLC1，后因其具有细胞粘附的功能而命名为CADM1（cell adhesion molecule 1）。TSLC1是由442个氨基酸组成的一种膜蛋白，在细胞粘附、突触形成、肿瘤抑制等功能中发挥重要作用。最近几项研究^[3-5]^同时表明敲除TSLC1可以引起雄性小鼠不育，Evi Michels等^[6]^报道TSLC1很可能是神经母细胞瘤的抑癌基因，最新的一项研究^[7]^表明TSLC1是静脉血栓形成的危险因子。迄今为止，对*TSLC1*研究最多的是其在多种肿瘤中发挥抑癌基因的功能及其表达下调的机制，如TSLC1在肺癌、前列腺癌、肝癌、胰腺癌、鼻咽癌、乳腺癌、宫颈癌、脑膜瘤等组织中表达明显下降或失活，被认为是一种抑癌基因^[8-22]^。在对TSLC1在多种肿瘤中表达下调原因的探索^[10]^中发现，突变失活不是其表达下调的主要原因，而TSLC1启动子区甲基化是一个频发事件，与TSLC1的表达下调呈相关性，用去甲基化试剂5-氮-2-脱氧胞苷（5-Aza-2-deoxycytidine, 5-Aza-dC）处理肿瘤细胞系后，TSLC1的表达水平可恢复正常，这些结果均提示TSLC1启动子区的甲基化可能是导致TSLC1表达下调的主要原因。为了明确探讨肺癌细胞系中TSLC1的表达水平及其启动子区的甲基化水平，本研究采用RTPCR及Real-time PCR方法检测TSLC1在人正常肺组织和3株肺癌细胞系中的表达水平，并运用bisulfite sequencing方法对TSLC1启动子区的甲基化水平进行检测，根据去甲基化试剂处理肺癌细胞系前后TSLC1的表达变化，判断TSLC1在肺癌细胞系中的表达水平是否与其启动子区的甲基化水平呈相关性。

## 1 材料与方法

### 1.1 材料

#### 1.1.1 组织与细胞系

人正常肺组织由北京医院胸外科提供，3株肺癌细胞系A549、NCI-H446、Calu-3及100×双抗、0.25%胰酶购自中国医学科学院基础医学研究所细胞中心，细胞培养用培养基McCOy-5A、RPMI-1640、MEM/NEAA及胎牛血清购自Gibco公司。

#### 1.1.2 试剂、耗材及引物

RNA抽提用Trizol购自Invitrogen公司，逆转录酶（RT酶）购自Biolab公司，Taq酶、Realtime PCR反应试剂购自TaKaRa公司。亚硫酸氢盐、氢醌及5-Aza-dC购自Sigma公司。基因组提取试剂盒购自QIAGEN公司，DNA片段回收试剂盒、pGEM-T载体购自Promega公司。文中所用引物由Invitrogen公司合成，引物序列详见表 1。

**1:** 本研究所用引物序列 The primer sequences used in this study

Methods		Primers	
Semiquantitative	TSLC1	Forward: 5’	-CTAGAAGTACAGTATAAGCCACAAG-3’
		Reverse: 5’	-AGTGAAGTATGTACCTTTATGTCTG-3’
RT-PCR	GAPDH	Forward: 5’	-ACCACAGTCCATGCCATCAC-3’
		Reverse: 5’	-TCCACCACCCTGTTGCTGTA-3’
Real-time PCR	TSLC1	Forward: 5’	- CCCCAGCCTGTGATGGTAA-3’
		Reverse: 5’	-GGATAGTTGTGGGGGGATCGTA-3’
	GAPDH	Forward: 5’	-CATCCATGACAACTTTGGTATC-3
		Reverse: 5’	-CCATCACGCCACAGTTTC-3

### 1.2 方法

#### 1.2.1 细胞培养

人肺癌细胞系用含10%胎牛血清和双抗（青霉素、链霉素各100 U/mL）的培养基，在37 ℃、5%CO_2_的孵箱中培养，根据实验需要收取细胞。

#### 1.2.2 5-Aza-dC处理肺癌细胞系

5-Aza-dC粉末用DMSO配置成20 mmol/L的母液，分装后存至-70 ℃。药物处理前1天接种细胞于60 mm培养皿中，接种细胞数以第2天细胞长至密度约20%为宜。第2天细胞换取新培养基，并向培养基中加入5-Aza-dC至终浓度10 μmol/L，此后每天换液加药1次，连续药物处理5天后，收取细胞进行实验。

#### 1.2.3 RT-PCR及Real-time PCR检测

按Invitrogen公司的说明书用Trizol一步法提取组织或细胞RNA，测量RNA浓度后，按Biolab公司说明书用RT酶将RNA反转录成cDNA。半定量RT-PCR反应条件为：94 ℃预变性5 min；94 ℃、30 s，55 ℃、30 s，72 ℃、35 s，反应25个循环；72 ℃充分延伸10 min，4 ℃终止反应，产物用2%琼脂糖进行电泳。Real-time PCR反应条件为94 ℃预变性10 s，94 ℃变性5 s，60 ℃退火34 s，进行45个循环后终止，结果运用Realtime PCR仪自带软件进行分析。

#### 1.2.4 Bisulfite sequencing

实验步骤按文献^[23]^进行，修饰后genome用作PCR模板。回收后的PCR产物连接到pGEM-T载体上，每种样品挑选11个阳性克隆送测序。

### 1.3 CpG岛分析软件及统计学处理

运用欧洲生物信息学研究所（EBI）提供的软件（http://www.ebi.ac.uk/Tools/emboss/cpgplot/）分析*TSLC1*基因5’非编码区的CpG岛。所有实验至少重复3次，实验数据以统计学分析软件SPSS 12.0进行统计处理。采用*Student’s t-test*比较结果，*P* < 0.05为差异具有统计学意义。

## 2 结果

### 2.1 TSLC1在正常肺组织和肺癌细胞系中的表达谱

半定量RT-PCR及Real-time PCR检测结果均表明，TSLC1在正常肺组织及肺癌细胞系A549中有表达，而在其它2株肺癌细胞系NCI-H446及Calu-3中表达明显下降或失活（图 1A，图 1B）。

**1:**
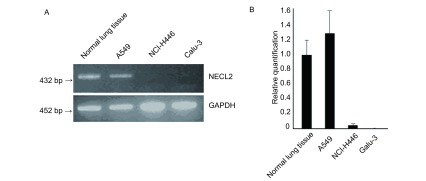
TSLC1在正常人肺组织及肺癌细胞系中的表达谱。半定量RT-PCR方法（A）及Real-time PCR方法（B）检测TSLC1在人正常肺组织及3株肺癌细胞系中的表达水平，GAPDH作为内对照。 Expression pattern of TSLC1 in human normal lung tissue and lung cancer cell lines. Expression levels of TSLC1 in human normal lung tissue and three lung cancer cell lines by semi-quantitative RTPCR (A) and Real-time PCR (B), GAPDH used as endogenous control.

### 2.2 *TSLC1*基因5’非编码区CpG岛分布

将*TSLC1*基因5’非编码区约3 500 bp序列在CpG岛分析软件中进行分析，发现在所分析的序列中存在一个CpG岛，它主要分布于翻译起始位点ATG附近约800 bp的区域内（图 2）。

**2:**
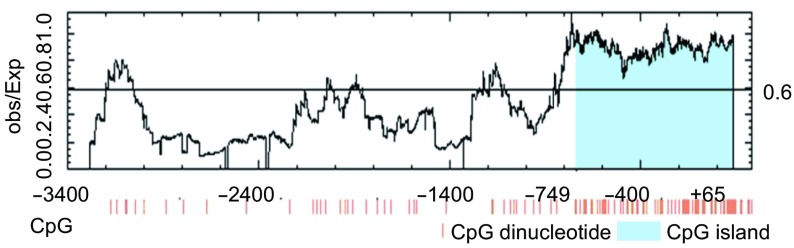
*TSLC1*基因5'非编码区的CpG岛分布。图下方的每条竖线代表一个CpG二核苷酸，蓝色区域代表CpG岛，翻译起始位点的“A”定义为+1。 The distribution of CpG island in 5' non-coding region of *TSLC1* gene. Each vertical short stick at the bottom of figure indicates one CpG dinucleotide, the blue region represents the CpG island and the translation start site "A" as +1.

### 2.3 TSLC1启动子区在正常肺组织和肺癌细胞系中的甲基化谱式

我们选取文献报道的与TSLC1表达下调密切相关的序列（TSLC1启动子区-556 bp到-336 bp之间的序列，翻译起始位点的“A”定义为+1）进行bisulfite sequencing检测，结果表明被检测的TSLC1启动子区在正常肺组织和A549细胞中呈非甲基化状态（图 3A，图 3B），而在NCI-H446及Calu-3细胞中呈高甲基化状态（图 3C，图 3D）。

**3:**
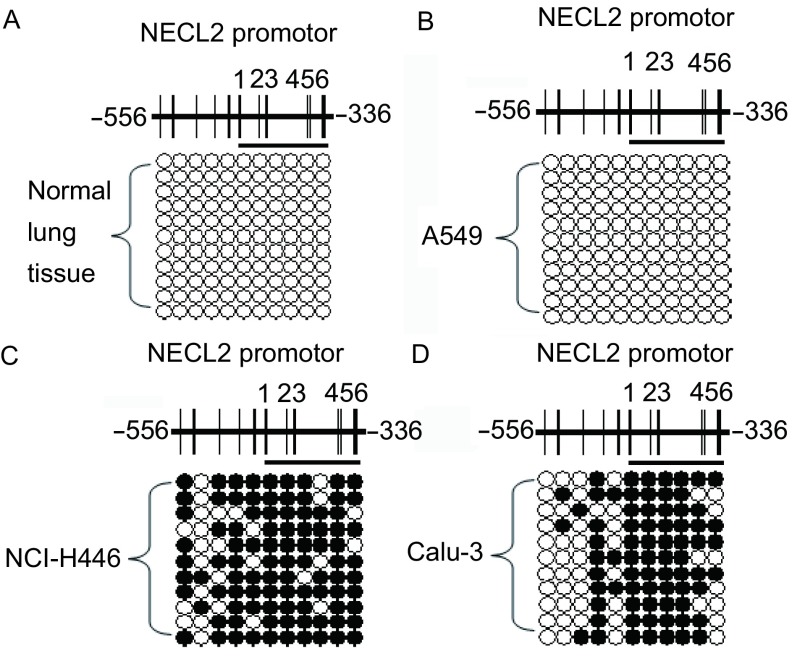
TSLC1在1例正常肺组织和3株肺癌细胞系中的甲基化谱式。A、B、C、D分别代表正常肺组织、A549细胞、NCI-H446细胞和Calu-3细胞中TSLC1启动子区的甲基化模式。每个图顶端的每条竖线代表一个CpG二核苷酸，被检测的TSLC1启动子区位于*TSLC1*基因组的-556 bp到-336 bp处，翻译起始位点的“A”定义为+1。被检测的*TSLC1*启动子区共包括11个CpG二核苷酸，其中标记为1号-6号的CpG位点是文献报道的重点检测位点。黑色圆圈表示发生甲基化的CpG；白色圆圈表示未发生甲基化的CpG。 Methylation pattern of TSLC1 in one human normal lung tissue and three lung cancer cell lines. A, B, C, D represented the methylation pattern of *TSLC1* promoter in normal lung tissue, A549, NCI-H446 and Calu-3 cell lines, respectively. Each vertical short stick at the top of each figure indicates one CpG dinucleotide, and *TSLC1* promoter region detected is located from -556 bp to -336 bp with the translation start site "A" as +1. Eleven CpG sites were included in this fragment and CpG sites which are marked from 1 to 6 are very important according to the papers. Black dot means methylated CpG while white dot means non-methylated CpG.

### 2.4 5-Aza-dC处理癌细胞前后TSLC1的表达

10 μmol/L的5-Aza-dC处理肺癌细胞系前后的Real-time PCR检测结果显示，5-Aza-dC处理A549细胞后TSLC1的表达未明显提高（图 4A），而5-Aza-dC处理NCI-H446和Calu-3细胞后TSLC1的表达得到明显提高（图 4B，图 4C）。

**4:**
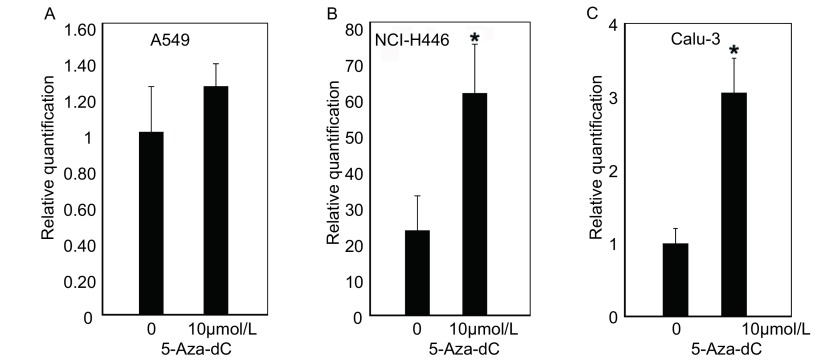
TSLC1在5-Aza-dC处理肺癌细胞系前后的表达谱。A、B、C分别代表TSLC1在5-Aza-dC处理A549细胞、NCI-H446和Calu-3细胞前后的表达水平；与对照组相比，*P < 0.05。 Expression pattern of TSLC1 in lung cancer cell lines before and after the treatment of 5-Aza-dC. A, B, C represented the expression level of TSLC1 in A549, NCI-H446 and Calu-3 cell lines before and after the treatment of 5-Aza-dC, respectively; *P < 0.05 compared with control.

## 3 讨论

人*TSLC1*为中国医学科学院基础医学研究所袁建刚教授实验室首次分离克隆的基因，定位于染色体11q23.12，编码一种人体组织广泛表达的由442个氨基酸组成的跨膜糖蛋白，属于免疫球蛋白超家族一员。TSLC1作为抑癌基因，在多种肿瘤中表达下降或失活，研究表明突变失活不是其表达下调的主要原因，而TSLC1启动子区甲基化是一个频发事件，与TSLC1的表达下调呈相关性。本研究应用RT-PCR、Real-time PCR及bisulfite sequencing方法检测了TSLC1在3种肺癌细胞中的表达与其启动子区甲基化的关系。结果显示TSLC1在正常肺组织和A549细胞中表达，而在NCI-H446和Calu-3细胞中表达缺失。运用软件对人*TSLC1*启动子区进行分析表明，*TSLC1*启动子区存在一个CpG岛，因此我们运用bisulfite sequencing方法检测了正常肺组织和肺癌细胞系中TSLC1启动子区的甲基化状态，结果表明在表达TSLC1的正常肺组织和A549细胞中，其启动子区未发生甲基化，而在TSLC1缺失表达的NCI-H446及Calu-3细胞中，其启动子区发生了高甲基化。我们用甲基化转移酶抑制剂5-Aza-dC处理肺癌细胞，用Real-time PCR方法检测处理前后TSLC1的表达水平，结果表明5-Aza-dC处理A549细胞前后，TSLC1的表达水平无明显变化，而5-Aza-dC处理NCI-H446及Calu-3细胞后，TSLC1的表达水平明显提高。上述结果具有很好的关联性，5-Aza-dC处理启动子未发生甲基化、表达TSLC1的肺癌细胞后，TSLC1的表达水平无明显改变，但5-Aza-dC处理启动子发生高甲基化、TSLC1缺失表达的肺癌细胞后，TSLC1的表达水平明显提高。这说明在TSLC1缺失表达的肺癌细胞中，TSLC1启动子的甲基化是引起其表达失活的机制之一。

在本研究中，我们运用RT-PCR及Real-time PCR方法检测了TSLC1转录水平的表达，因实验进行时尚无商品化TSLC1抗体，自制的TSLC1抗体特异性差，故而无法从蛋白水平检测其表达，此外，5-Aza-dC通过抑制mRNA转录进而影响蛋白表达，而且大多数研究均从RNA水平检测5-Aza-dC处理前后目的基因的变化，因此本研究结果具有一定的说服力，若有条件从蛋白水平检测TSLC1的表达及药物处理前后的变化会更有意义。该研究的前期实验结果表明，TSLC1在人正常肺组织中高表达，而且其它研究也表明TSLC1在正常肺组织中高表达，在肺癌组织中表达缺失，因此本研究选取取材方便的正常肺组织作为阳性对照以判断肺癌细胞系是否表达TSLC1。本文是从细胞水平对TSLC1的表达缺失与甲基化关系进行了初步探讨，若能选取正常肺细胞作为阳性对照，本文的前后一致性会更强。我们选取的细胞系中，A549及Calu-3是肺腺癌细胞系，NCI-H446是小细胞肺癌细胞系，3种细胞系中TSLC1的表达及甲基化谱不一致，可能与不同来源的肺癌细胞的生物学行为有关。3种细胞均具有致瘤能力，但它们在肿瘤发生过程中表现出的生物学行为尚不清楚，有待深入研究。

表观遗传调控是目前基因组学研究的热点，包括DNA甲基化、组蛋白乙酰化、泛素化及miRNA调控等。组蛋白去乙酰化也是导致基因表达沉默的机制之一^[24]^，而且常与DNA甲基化协同作用抑制基因表达^[25]^。miRNA是目前研究基因表达调控的热门领域，通过靶向作用于目的基因而抑制基因的转录^[26]^。本文从DNA甲基化入手研究其与TSLC1表达的关系，因为已有研究证实在多种肿瘤中DNA高甲基化是引起TSLC1表达失活的主要机制，因此本文结果从这一方面证明DNA高甲基化是抑制TSLC1在肺癌细胞中表达失活的机制之一。基因表达调控是一个很复杂的网络，是否还有其它机制引起TSLC1在肺癌中的表达缺失尚需深入研究。

虽然我们的实验结果表明*TSLC1*启动子区的甲基化可导致基因表达的明显下降，但*TSLC1*启动子区甲基化造成转录失活的具体机制仍不清楚，有待后续研究。例如，我们可以通过电泳迁移率变动分析实验或染色质免疫共沉淀实验来探寻与之相结合的甲基化CpG结合蛋白，以此寻找线索。此外，导致肿瘤中*TSLC1*启动子区高甲基化的机制亦不清楚，亟待我们进一步研究。
